# Research on Hysteresis of Piezoceramic Actuator Based on the Duhem Model

**DOI:** 10.1155/2013/814919

**Published:** 2013-06-13

**Authors:** Miaolei Zhou, Jingyuan Wang

**Affiliations:** College of Communication Engineering, Jilin University, Changchun 130012, China

## Abstract

To improve the modeling accuracy of piezoceramic actuator in the precision positioning system, the Duhem hysteretic model of the piezoceramic actuator was proposed. The paper used the polynomial function to approach the piecewise continuous function and *f*(*v*) and *g*(*v*) in the Duhem model, adopted recursive least squares algorithm and gradient correction algorithm to identify parameter **α**, polynomial coefficients of *f* and *g* in the Duhem model, and established the nonlinear parametric model of the piezoceramic actuator. Contrasting the simulation results of recursive least squares algorithm and gradient correction algorithm, the modeling accuracy is 0.24% when adopting the recursive least squares algorithm, and the modeling accuracy is 0.11% when adopting the gradient correction method. The result showed that the gradient correction algorithm could meet the modeling accuracy better, and the structure of the algorithm is simple, adaptable, and easy to implement.

## 1. Introduction

The piezoceramic actuator is a kind of ideal drive elements in the microdisplacement technology currently and has the advantage of high positioning accuracy, large driving force, and fast response speed. Since the hysteresis, nonlinear, and creep resistance, inherent the piezoceramic actuator, the repeatability and accuracy of microdisplacement mechanism decreased, and the transient response speed becomes slower. [1–8] To decrease the impact of the nonlinearity and obtain better performance of the piezoceramic actuator, many researchers carried on an investigation in modeling and controlling of hysteresis nonlinear system. [9–15]  Al Janaideh et al. [16] analyzed the inverse model of generalized Prandtl-Ishlinskii (PI) model to compensate the hysteresis nonlinearities of smart actuators. The experimental results verified that the inverse model of generalized PI model could be conveniently applied as a feedforward compensator and saturated hysteresis in magnetostrictive and SMA actuators. Dong and Tan [11] proposed a modified PI modeling method for rate-independent hysteresis in piezoelectric actuators and introduced a generalized backlash operator as the elementary operator into the model. By applying the proposed method, the hysteresis in the piezoelectric actuators and ultrasonic motor are illustrated, respectively. Hamimid et al. [17] proposed the minor hysteresis loops model based on parameters scaling of the modified Jiles-Atherton model by using judicious expressions. The proposed model had been applied for 3.2% Fe-Si nonoriented magnetic sheet, and the result showed the expected behavior when the flux density level was low. Chwastek [18] used the dynamic Takacs model to describe hysteresis loops in a thick nonoriented steel sheet and achieved the dynamic extension by using an additional component of the effective field. The experiment obtained a satisfactory agreement between the measured and the modeled hysteresis loops. Baghel and Kulkarni [19] proposed a hybrid technique to solve the parameter identification problem based on the Jiles-Atherton hysteresis model. And the proposed technique is flexible enough to incorporate improved GA and LM algorithms. Zirka et al. [20] analyzed the physical assumptions under the static and dynamic Jiles-Atherton (JA) hysteresis models. This led to the using in the model of a misleading entity resembling the coenergy instead of the actual energy. It is necessary to take measures to avoid this nonphysical feature; therefore the JA static model is reserved for applying in circuit simulators.

The Duhem model is a kind of differential hysteresis model, proposed by Duhem and Stefanini in 1897 [21]. The Duhem model has the explicit function expression and is the function of input signal derivative; the output of model is related to the rate of input signal. The model is a kind of dynamic models [10], conforms the dynamic characteristic of hysteresis nonlinear in the actual intelligent materials, and could describe the hysteresis nonlinear precisely. However it is difficult to obtain the parameter *α*, coefficients of *f* and *g* in the Duhem model, and it would be the obstacles for application of Duhem model.

## 2. Parameters Identification of the Hysteresis Model

The Duhem has an explicit differential expression, through adjusting the parameter *α*, coefficients of *f* and *g* of the Duhem model; different hysteresis characteristics could be reflected; while identifying the parameters of the Duhem model accurately, the hysteresis model of the piezoceramic actuator could be obtained [2]. 

The differential function of the Duhem model is

(1) $$\begin{array}{c} \frac{dw}{dt}=\alpha \left|\frac{dv}{dt}\right|\left[f\left(v\right)-w\right]+\frac{dv}{dt}g\left(v\right), \end{array}$$

where *α* is constant, *v* is the input voltage, *w* is output displacement, *f*(*v*) and *g*(*v*) are piecewise continuous functions. 

*C*[*a*, *b*] represents the set of all continuous functions defined in the closed interval [*a*, *b*], to the arbitrary *f*(*v*) and *g*(*v*) in the *C*[*a*, *b*]; ||*f*−*h*||_*∞*_ = sup⁡_*a*≤*v*≤*b*_|*f*(*v*) − *h*(*v*)| represents the distance between them [2]. Letting *f* ∈ *C*[*a*, *b*], to the arbitrary given *ε* > 0, the polynomial existed and the following equation holds:

(2) $$\begin{array}{c} {||f-h||}_{\infty }=\underset{a\leq v\leq b}{\sup}\left|f\left(v\right)-h\left(v\right)\right|\leq \varepsilon . \end{array}$$

To the arbitrary given *f*(*v*) ∈ *C*[*a*, *b*] and approximation precision, there has an algebraic polynomial

(3) $$\begin{array}{c} h\left(v\right)=a_{0}+a_{1}v+a_{2}v^{2}+\cdots +a_{m}v^{m}, \end{array}$$

where *m* is natural number and ||*f*−*h*||_*∞*_ ≤ *ε*. 

When accuracy *ε* > 0, the order of *f*(*v*) and *g*(*v*) is *m* and *n*, respectively; the polynomials are as follows:

(4) $$\begin{array}{c} f\left(v\right)=f_{0}+f_{1}v+f_{2}v^{2}+\cdots +f_{m}v^{m}=\sum_{i=0}^{m}f_{i}v_{i}^{i},\\ g\left(v\right)=g_{0}+g_{1}v+g_{2}v^{2}+\cdots +g_{n}v^{n}=\sum_{j=0}^{n}g_{j}v_{j}^{j}. \end{array}$$

Substituting (4) into (1),

(5) $$\begin{array}{c} \frac{dw}{dt}=\left|\frac{dv}{dt}\right|\left[\alpha f\left(v\right)-\alpha w\right]+\frac{dv}{dt}g\left(v\right). \end{array}$$

And (5) could be transformed into

(6) $$\begin{array}{c} \frac{dw}{dt}=\left|\frac{dv}{dt}\right|\left[\alpha \sum_{i=0}^{m}f_{i}v_{i}^{i}-\alpha w\right]+\frac{dv}{dt}\sum_{j=0}^{n}g_{j}v_{j}^{j}. \end{array}$$

As the input voltage *v*, output displacement *w*, and *dv*/*dt*, *dw*/*dt* are measurable, while identifying the parameters *α*, *f*_*i*_, and *g*_*j*_ accurately, the parameterized model of the Duhem model could be obtained. 

Letting *V*(*k*) = |*v*(*k*) − *v*(*k* − 1)|, *W*(*k*) = *v*(*k*) − *v*(*k* − 1), *Y*(*k*) = *w*(*k*) − *w*(*k* − 1), *k* = 2,3,…, the dynamic discretization Duhem model of the system is

(7) $$\begin{array}{c} Y\left(k\right)=V\left(k\right)\cdot \left[\alpha \sum_{i=0}^{m}f_{i}v{\left(k\right)}^{i}-\alpha w\left(k\right)\right]+W\left(k\right)\cdot \sum_{j=0}^{n}g_{j}v{\left(k\right)}^{j}, \end{array}$$

where *v*(*k*) is the input voltage of the system at time *k*, *W*(*k*) is the output displacement at time *k*.

Letting *Y*(*k*) = *φ*(*k*)^*T*^ × *θ*, *φ*(*k*) is the data vectors of the input voltage, *θ* is the identified parameter vector. That is,

(8) φ(k)T=[V(k),V(k)v(k),…,V(k)v(k)m,−V(k)w(k), W(k),W(k)v(k),…,W(k)v(k)n]             φ(k)T∈R1×(m+n+3),θ=[f0,f1,f2,…,fm,α,g0,g1,g2,…,gn]               θ∈R1×(m+n+3).

Let

(9) $$\begin{array}{c} J\left(\theta \right)=\sum_{k=1}^{\infty }{\left[e\left(k\right)\right]}^{2}=\sum_{k=1}^{\infty }\left\{{\left[y\left(k\right)-\varphi {\left(k\right)}^{T}\times \theta \right]}^{2}\right\}, \end{array}$$

that is,

(10) $$\begin{array}{c} e\left(k\right)=Y\left(k\right)-\varphi {\left(k\right)}^{T}\times \theta . \end{array}$$

The target of the parameter identification is to obtain the value of the parameter *θ* when the function is the minimum.

Applying the recursive least squares algorithm to recursive equations (11), (12), and (13), the identification parameters are

(11) $$\begin{array}{c} \overset{\wedge }{\theta }\left(k\right)=\;\overset{\wedge }{\theta }\left(k-1\right)+K\left(k\right)\left[y\left(k\right)-\varphi {\left(k\right)}^{T}\overset{\wedge }{\theta }\left(k-1\right)\right], \end{array}$$

(12) $$\begin{array}{c} K\left(k\right)=\frac{P\left(k\right)\varphi \left(k+1\right)}{1+\varphi {\left(k\right)}^{T}P\left(k-1\right)\varphi \left(k\right)}, \end{array}$$

(13) $$\begin{array}{c} P\left(k\right)=\left[1-K\left(k\right)\varphi {\left(k\right)}^{T}\right]P\left(k-1\right). \end{array}$$

Equation (11) is the parameterized model.

The recursive equation of the gradient correction parameter estimate is

(14) $$\begin{array}{c} \overset{\wedge }{\theta }\left(k\right)=\;\overset{\wedge }{\theta }\left(k-1\right)+R\left(k\right)\varphi \left(k\right)\varepsilon \left(k\right),\\ \varepsilon \left(k\right)=\left[y\left(k\right)-\varphi {\left(k\right)}^{T}\overset{\wedge }{\theta }\left(k-1\right)\right], \end{array}$$

where *R*(*k*) is the weight matrix, the effect of the weight is to control the influence of the input component.

Let

(15) $$\begin{array}{c} R\left(k\right)=c\left(k\right)\mathrm{diag}\left[\Lambda_{1}\left(k\right),\Lambda_{2}\left(k\right),\ldots ,\Lambda_{N}\left(k\right)\right]. \end{array}$$

If the element of the weight matrix meets the following conditions:

(1) 0 < Λ_*L*_ ≤ Λ_*i*_(*k*) ≤ Λ_*H*_  (*i* = 1,2,…, *N*), Λ_*L*_ and Λ_*H*_ are the determined upper and lower bound values;
(2) at least one Λ_*m*_(*k*) existed for *N*, that
  (16) $$\begin{array}{c} \frac{\Lambda_{m}\left(k\right)-\Lambda_{m}\left(k+1\right)}{\Lambda_{m}\left(k\right)}\geq \frac{\Lambda_{i}\left(k\right)-\Lambda_{i}\left(k+1\right)}{\Lambda_{i}\left(k\right)} \end{array}$$
  or
  (17) $$\begin{array}{c} \frac{\Lambda_{m}\left(k+1\right)}{\Lambda_{m}\left(k\right)}\leq \frac{\Lambda_{i}\left(k+1\right)}{\Lambda_{i}\left(k\right)}; \end{array}$$
(3) 0 < *c*(*k*) < 2/∑_*i*=1_^*N*^Λ_*i*_(*k*)*φ*_*i*_^2^(*k*);
(4) $\overset{\sim }{\theta }(k)$ and *φ*(*k*) are disjoint; $\overset{\sim }{\theta }\left(k\right)=\theta_{0}-\overset{\wedge }{\theta }\left(k\right)$,

Then regardless of the initial value of the parameter estimate, the parameter estimated value is always wide range asymptotic convergence, that is

(18) $$\begin{array}{c} \underset{k\to 0}{\lim}\overset{\wedge }{\theta }\left(k\right)=\theta_{0}. \end{array}$$

## 3. Parameters Identification Simulation and Modeling of the Duhem Model

To verify the accuracy of the algorithms for the Duhem model identification, the paper applied recursive least squares algorithm and gradient correction algorithm to identify the parameters of the Duhem model based on MATLAB simulation software, respectively, and contrasted the influence of the two identification algorithms to the model modeling accuracy.

### 3.1. Identification of Recursive Least Squares Algorithm

In the experiment, the order of the polynomial *f*(*v*), *g*(*v*) is *m* = 3, *n* = 2, respectively; that is,

(19) $$\begin{array}{c} f\left(v\right)=f_{0}+f_{1}v\left(k\right)+f_{2}v{\left(k\right)}^{2}+f_{3}v{\left(k\right)}^{3},\\ g\left(v\right)=g_{0}+g_{1}v\left(k\right)+g_{2}v{\left(k\right)}^{2}. \end{array}$$

**Figure 1:**
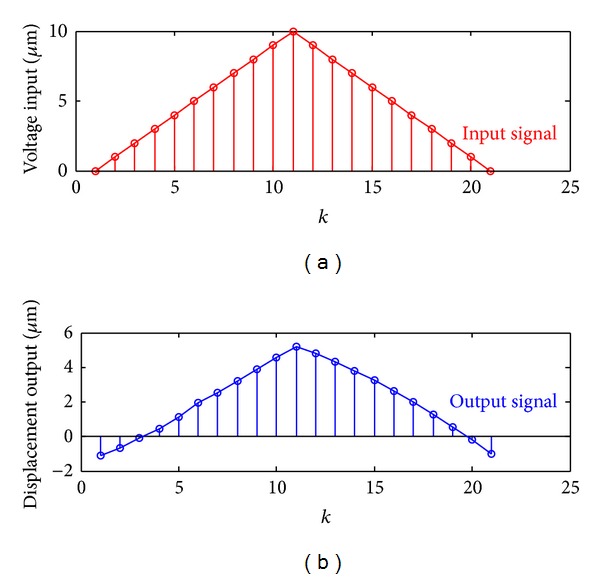
Given input-output curves.

Figure 1 is the given input-output curves; there are 21 sets of data totally. The red line represents the voltage input signal, and the blue line represents the displacement output signal. Under recursive least squares algorithm, the identification result is

(20) α=0.0874,f(v)=−0.015+0.006v(k)+5.57×10−6v(k)2−8.6×10−8v(k)3,g(v)=0.053−8.316×10−4v(k)+8.195×10−6v(k)2.

**Figure 2:**
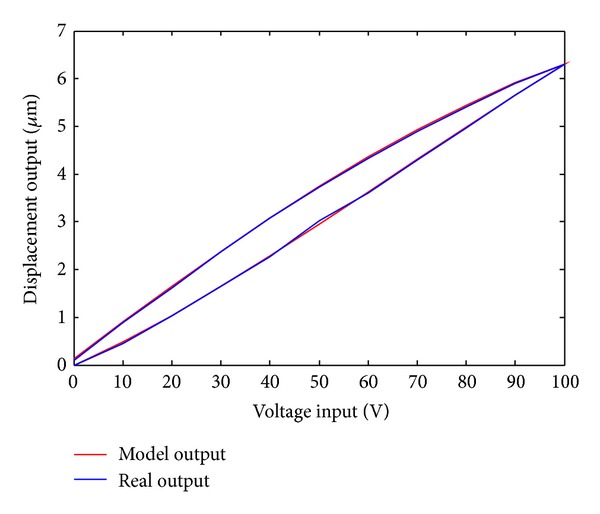
Input-output hysteresis curves of Duhem model.

Utilizing the parameter identification data, the hysteresis curve of the model is shown in Figure 2. The red and blue curves represent input-output hysteresis curve of the Duhem model and actual input-output hysteresis curve, respectively.

**Figure 3:**
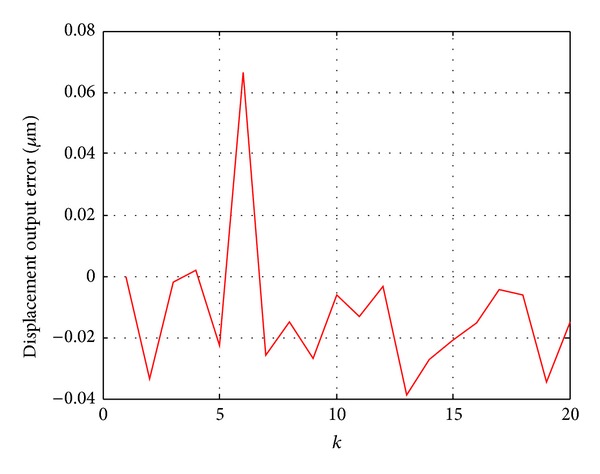
Error curve between the actual output and model output.

Figure 2 showed that the output of the Duhem model and the actual output data are basically consistent.  Figure 3 is the relative error curve between the model output and actual output. It can be seen that the maximum error is 0.066 *μ*m. The result of the experiment verified the validity of the recursive least squares algorithm.

### 3.2. Gradient Correction Algorithm

The data of the input and output is shown in Figure 1; under the gradient correction algorithm, Λ_*i*_(*k*) is as follows:

(21) $$\begin{array}{c} \mathrm{diag}\Lambda_{i}\left(k\right)=\left[1,\frac{1}{3},\frac{1}{3^{2}},\frac{1}{3^{3}},1,1,\frac{1}{3},\frac{1}{3^{2}}\right]. \end{array}$$

**Figure 4:**
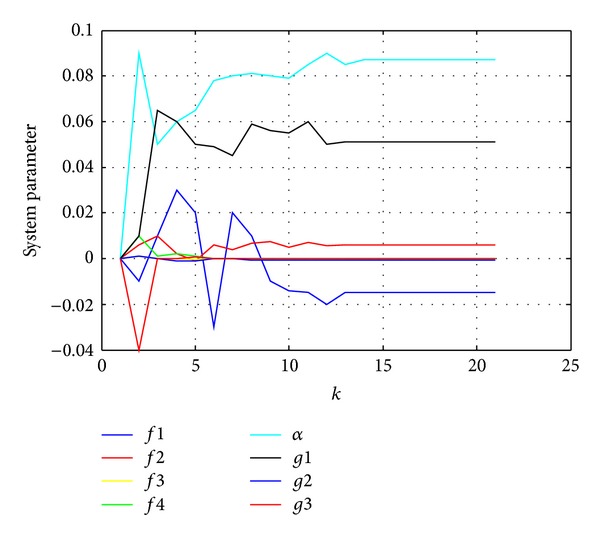
Parameter identification curves of the gradient correction algorithm.

The parameter identification result is shown in Figure 4. It can be seen form Figure 4 that the identification parameters tend to be stable when recursiving to *k* = 6; the parameter identification results are as follows:

(22) α=0.087,f(v)=−0.0149+0.0061v(k)+5.5649×10−6v(k)2−8.6096×10−8v(k)3,g(v)=0.051−8.3166×10−4v(k)+8.1960×10−6v(k)2.

**Figure 5:**
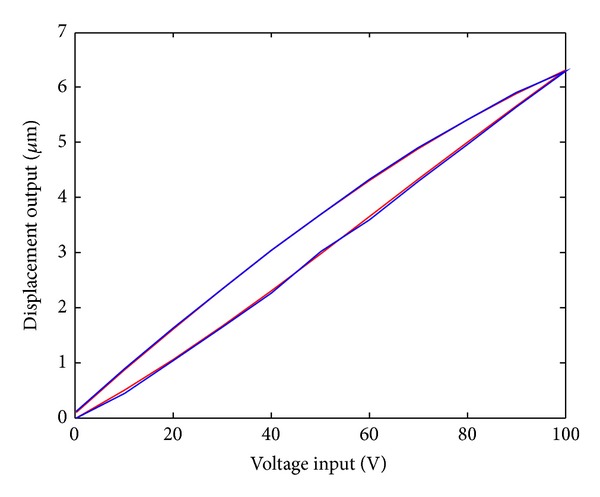
Input-output hysteresis curves of Duhem model.

**Figure 6:**
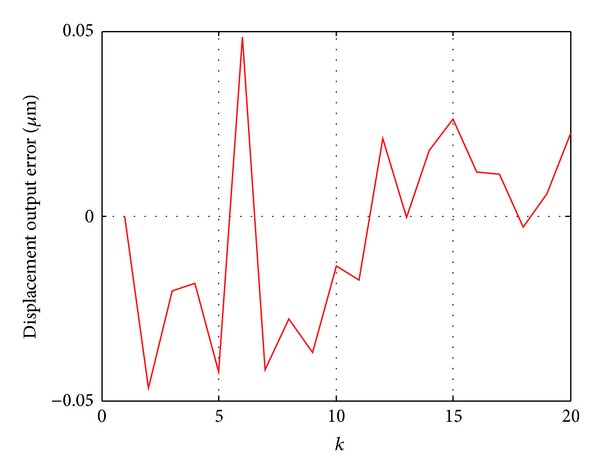
Error curve between the actual output and model output.

Utilizing the gradient correction parameter identification results, the model hysteresis curve is shown in Figure 5, The red and blue curves represent input-output hysteresis curve of the Duhem model and real input-output hysteresis curve, respectively. The error curve between the system output and model output is shown in Figure 6. It can be seen from Figure 6 that the maximum is 0.048 *μ*m. The result also verified the validity of the gradient correction algorithm.

The identification parameters of the two algorithms are shown in Table 1. And we show part of the relative errors contrast results under the two algorithms in Table 2. It can be seen from Table 1 that the relative errors between the real output and model output under the recursive least squares algorithm could reach 0.24%, the mean square deviation of the error is 0.0263, and the maximum error is 0.066 *μ*m; in contrast, the relative errors between the actual output and model output under the gradient correction algorithm could reach 0.11%, the mean square deviation of the error is 0.0222, and the maximum error is 0.048 *μ*m.

## 4. Conclusion

The paper utilized the polynomial to approach the piecewise continuous functions *f* and *g* of the Duhem model, adopted the recursive least squares and gradient correction algorithm, respectively, to identify the parameter *α*, coefficients of *f* and *g* of the Duhem model, and applied the identified parameters to model the Duhem model. The experiment results showed that the modeling accuracy of the recursive least squares algorithm could reach 0.24%, the mean square deviation of the error is 0.0263; the modeling accuracy of the gradient correction algorithm could reach 0.11%, the mean square deviation of the error is 0.0222. The results of the experiment certified validity of the recursive least squares algorithm and gradient correction algorithm. Contrasting with the least squares algorithm, the gradient correction algorithm is adaptable and suitable for engineering. Applying the gradient correction algorithm, the Duhem model could be established more precisely and lay the foundation for the further control research of the piezoceramic.

## Figures and Tables

**Table 1:** Identification parameters of two algorithms.

Identification parameters	Recursive least squares algorithm	Gradient correction algorithm
*f*_1_	−0.015	−0.0149
*f*_2_	0.006	0.0061
*f*_3_	5.57*e* − 6	5.5649*e* − 6
*f*_4_	−8.6*e* − 8	−8.6096*e* − 8
*α*	0.0874	0.0870
*g*_1_	0.053	0.051
*g*_2_	−0.83*e* − 4	−0.83166*e* − 4
*g*_3_	8.1*e* − 6	8.196*e* − 6

**Table 2:** The relative errors of two algorithms.

*k*	Relative error (gradient correction algorithm)	Relative error (recursive least squares algorithm)
2	−0.0699	−0.0941
4	0.0012	−0.0110
6	0.0225	0.016
8	−0.0034	−0.0065
10	−0.0011	−0.0024
12	0.0021	0.0035
14	0.0055	0.0036
16	−0.0041	0.0086
18	−0.0026	0.0159
20	−0.0167	0.0259
Mean square deviation of the error	0.0222	0.0263
Maximum error	0.048 *μ*m	0.066 *μ*m
